# Evaluation of genetic susceptibility of common variants in SOX9 in patients with congenital talipes equinovarus in the Han Chinese population

**DOI:** 10.1186/s13018-020-01802-7

**Published:** 2020-07-23

**Authors:** Jian Li, Zhi Wang, Dongxu Feng, Wei Wang, Weilou Feng

**Affiliations:** 1 Department of Sports Medicine, HongHui Hospital, Xi’an Jiaotong University, Xi’an, Shaanxi China; 2 Department of Neonatology, Xi’an Children Hospital, Xi’an, Shaanxi China; 3 Department of Orthopaedic Trauma, HongHui Hospital, Xi’an Jiaotong University, Xi’an, Shaanxi China

**Keywords:** Congenital talipes equinovarus, *SOX9* gene, Single-nucleotide polymorphisms, Genetic association, Case-control study

## Abstract

**Background:**

Congenital talipes equinovarus (CTEV) is a common birth defect that causes severe deformities of one or both feet. Genetics have been proven to play a key role in the risk of CTEV. Our study aimed to evaluate the genetic susceptibility of common variants in the *SOX9* gene to CTEV in a Han Chinese population.

**Methods:**

In this study, we recruited 2,205 study participants, including 692 CTEV patients and 1513 healthy controls. A total of seven selected single-nucleotide polymorphisms (SNPs) within the *SOX9* gene were genotyped, and environmental variables, including maternal smoking and alcoholic drinking habits, were assessed. In addition, bioinformatics analyses were performed to explore the potential biological functions of the associated SNPs.

**Results:**

The SNP rs73354570 was identified to be significantly associated with the risk of CTEV (OR = 1.53, *P* = 2.11 × 10^−5^), and the C allele was associated with an increased risk of CTEV. A dose-dependent pattern could be observed in genotypic analyses. The OR for individuals with AC genotypes was 1.37 (95% CI 1.09–1.71), and the OR for individuals with CC homozygotes was 1.47 (95% CI 1.18–1.82). Further analyses identified that rs73354570 is located within a region of multiple binding proteins, including CEBPB and POLR2A, which suggested that this SNP was also part of genetic motifs that are found within several cell types.

**Conclusion:**

Our results provide evidence supporting the important role of the *SOX9* gene in the contribution to the risk of CTEV.

## Background

Congenital talipes equinovarus (CTEV), or clubfoot, is a common birth defect that occurs in 1 in every 1000 live births [1]. As a severe birth defect, patients with this disorder have difficulty walking because of severe deformities of one or both feet [1]. The etiology of CTEV is still unclear, but genetics have been proven to play a key role in the risk of CTEV [2–5]. In a family-based study, Gurnett et al. showed that clubfoot segregates in an autosomal dominant fashion with incomplete penetrance [6]. Previous genetic studies have identified several genes that are related to clubfoot, including *PITX1*, *TBX4*, and *MYH3*. The only previous genome-wide association study (GWAS) of clubfoot identified a significant single-nucleotide polymorphism (SNP) between *NCOR2* and *ZNF664*, and there was evidence suggesting that *FOXN3*, *SORCS1*, and *MMP7/TMEM123* were also associated with clubfoot [7].

*SOX9* encodes a transcription factor that can recognize and bind to the sequence of CCTTGAG. Early studies have shown that the products of *SOX9* can bind to *COL9A1* and regulate its transcription [8]. *COL9A1* encodes an α chain of type IX collagen. Early studies have shown that mice with a knockout of the Col9a1 gene do not produce any collagen IX polypeptides, which indicates that *COL9A1* is essential for the synthesis of type IX collagen. Recent studies have shown that variations in collagen IX-related genes can cause alterations in bone marrow hyperplasia and might therefore be related to articular cartilage-related diseases [8, 9]. Zhou et al. investigated the association between common SNPs of *COL9A1* and clubfoot in a small sample of individuals with Chinese Han ancestry, and a potentially related SNP was identified [10]. Based on these multiple lines of evidence connecting *SOX9* and *COL9A1*, we hypothesize that *SOX9* might be involved in the regulation of articular cartilage development. In a recent study, Wang et al. detected significant differences in the mRNA and protein expression levels of *SOX9* between idiopathic CTEV muscle samples and controls [11]. Nevertheless, no evidence has been identified to connect the genetic polymorphisms of *SOX9* and the risk of CTEV in the human population.

In this study, we aimed to investigate the potential genetic association between genetic polymorphisms of *SOX9* and the risk of CTEV based on thousands of study participants with Chinese Han ancestry. In addition to single marker-based association analyses, we also examined the effects of some environmental factors, including maternal smoking and alcoholic drinking habits. Gene-by-environment interactions were also explored.

## Methods

### Study participants

We recruited 692 CTEV patients and 1513 unrelated healthy controls from Honghui Hospital of Xi’an Jiaotong University and Xi’an Children Hospital from January 2012 to April 2017. All participants included in the study were randomly chosen genetically unrelated Han Chinese individuals. All patient diagnoses were confirmed by X-ray examinations. All controls without foot deformities were matched with patients by age and gender. The demographic and clinical characteristics of all participants were obtained from questionnaires and medical records. This study was performed in accordance with the ethical guidelines of the Helsinki Declaration of 1975 (revised in 2008) and was approved through the Ethics Committee of Honghui Hospital of Xi’an Jiaotong University. Written informed consent was obtained from all participants.

### SNP selection and genotyping

Tagged SNPs located within gene regions with minor allele frequency (MAF) > 0.01 in *SOX9* in 1000 Chinese Han genomes were chosen for genotyping. Algorithm Tagger integrated in Haploview [12] was used for SNP tagging, and the *r*^2^ criterion used for tagging was 0.8 for both gene regions. The *r*^2^ criterion was used for tagging. A total of 7 candidate SNPs were selected for genotyping in this study.

Genomic DNA was isolated from peripheral blood leukocytes according to the manufacturer’s protocol (Genomic DNA kit, Axygen Scientific, Inc., CA, USA). SNP genotyping was performed using the high-throughput Sequenom MassARRAY platform with iPLEX GOLD chemistry (Sequenom, San Diego, CA, USA) based on the manufacturer’s protocols. The results were processed using Sequenom Typer 4.0 software, and genotype data were generated from the samples [13]. The case and control sample results were blinded for quality control during genotyping processes [14], and 5% of samples were randomly processed with a concordance of 100%.

### Statistical methods

Genetic association analyses were performed at both genotypic and allelic levels using Plink v1.9 [15]. A linkage disequilibrium (LD) plot was made using Haploview [12]. In addition to ordinary genetic association analyses, we conducted gene-by-environment interaction analyses by logistic models. For gene-by-environment analyses, two environmental factors, i.e., maternal smoking and maternal alcoholic drinking, were included. The smoking and drinking status were recorded as their habit in general but not status during their pregnancy. Each of these seven selected SNPs was examined by pairing with each of the two environmental factors. Both maternal smoking and alcoholic drinking were coded as 0, 1, and 2, indicating never, occasionally, and often, respectively. Age and gender were included in the logistic models described above. Bonferroni corrections were applied to address multiple comparisons. In addition to statistical methods, we conducted bioinformatics analyses to explore the potential biological functions of our targeted SNPs. RegulomeDB, which annotates SNPs using ENCODE data, was used to investigate the potential regulatory roles of significant SNPs [16]. We also examined the potential eQTL patterns of those significant SNPs using GTEx [17]. Moreover, we investigated the gene-gene network of *SOX9* using the STRING database, which is a database of known and predicted protein-protein interactions.

## Results

### Demographic and clinical characteristics of our study participants

Of the patients included in this study, 8.1% had a family history of CTEV compared with 1.4% of the controls. There was a significant difference in this variable between the study groups (*P* < 0.001, Table 1). However, no significant differences were found in age, gender, maternal smoking, or maternal drinking between groups (Table 1).

**Table 1:** Demographic characteristics of CTEV patients and healthy controls

Characteristics	Subjects (*N* = 2205)		*P* value
	Patients	Controls	
Number	692	1513	–
Age (months), mean ± SD	46.72 ± 32.30	46.91 ± 33.34	0.99
Gender (male/female)	445/247	970/543	0.96
Family history (yes/no)	56/636	21/1492	< 0.001
Maternal smoking (yes/occasionally/never)	44/91/557	81/170/1262	0.25
Maternal drinking (yes/occasionally/never)	55/208/429	117/436/960	0.80
Talipes equinovarus (single/both)	411/281	––	–

*SD* standard deviation, *NA* not applicable. Chi-square tests were performed for qualitative variables, and *t* tests were conducted for quantitative variables

### Genetic associations signal

**Fig. 1:**
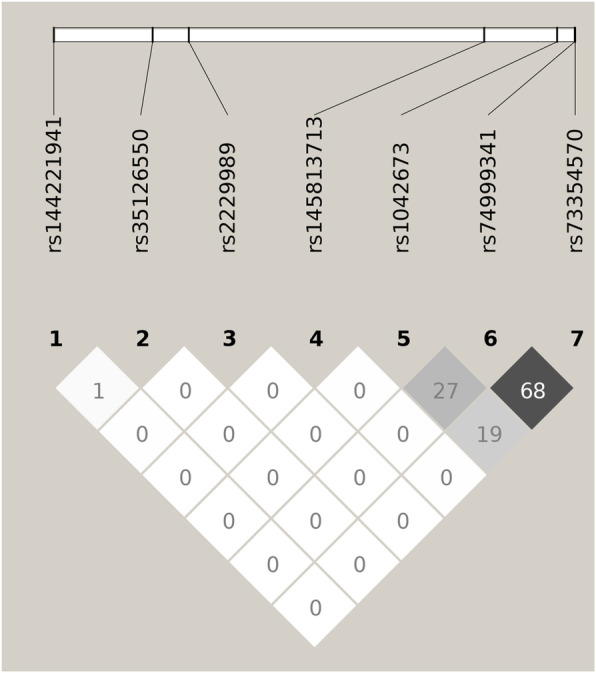
Linkage disequilibrium plot made by the genotyped SNPs. Values of *r*^2^ were showed in each cell

In our study, all seven SNPs selected were in Hardy-Weinberg equilibrium. Basic information, including MAF and the results of Hardy-Weinberg equilibrium tests for these 7 SNPs, is included in Table 2. Weak LD could be identified among these 7 SNPs (Fig. 1). Rs73354570 of *SOX9* was the only SNP identified to be significantly associated (odds ratio = 1.53, *P* = 2.11 × 10^−5^) with the disease status of CTEV (Table 3). The C allele was associated with an increased risk of CTEV. A dose-dependent pattern could be observed in genotypic analyses. The ORs for individuals with AC genotypes and CC homozygotes were 1.37 (with 95% confidence interval 1.09–1.71) and 1.47 (with 95% confidence interval 1.18–1.82), respectively. The only interaction signal that achieved nominal significance was identified between SNP rs73354570 and maternal alcoholic drinking habit (*P* = 0.02). However, this signal was no longer significant if we correct for multiple comparisons (threshold of *P* value is 0.007). The full results are summarized in Table 4.

**Table 2:** Genetic information of the selected SNPs

CHR	SNP	POS	ALLELE	FUNC	LOCI	MAF	HWE
17	rs144221941	72121913	C/T	Near-gene-5	*SOX9*	0.03	0.62
17	rs35126550	72122558	A/G	Intron	*SOX9*	0.49	0.92
17	rs2229989	72122794	C/T	Coding-synon	*SOX9*	0.03	0.41
17	rs145813713	72124722	A/G	Untranslated-3	*SOX9*	0.02	1.00
17	rs1042673	72125198	A/G	Untranslated-3	*SOX9*	0.23	0.94
17	rs74999341	72125313	C/T	Untranslated-3	*SOX9*	0.13	1.00
17	rs73354570	72125316	A/C	Untranslated-3	*SOX9*	0.11	0.23

CHR: chromosome; POS: position; FUNC: function; MAF: minor allele frequency;HWE: *P* values of Hardy-Weinberg Equilibrium tests performed in controls.

**Table 3:** Genetic association between genetic polymorphisms of *SOX9* and CTEV

CHR	SNP	POS	Genotypic analyses						Allelic Analyses					
			Genotypes	Cases	Controls	OR[95% CI]	*χ^*2*^	*P* value	Alleles	Cases	Controls	OR[95% CI]	*χ*^*2*^	*P* value
			TT	0	0									
17	rs144221941	72121913	CT	40	79	1.13[0.75–1.65]			T	40	79	1.11[0.75–1.63]		
			CC	652	1434	ref	–	0.61	C	1344	2947	ref	0.28	0.60
			AA	165	356	1.03[0.80–1.33]								
17	rs35126550	72122558	AG	346	753	1.03[0.83–1.27]			A	676	1465	1.02[0.90–1.16]		
			GG	181	404	ref	0.08	0.96	G	708	1561	ref	0.07	0.79
			TT	0	0									
17	rs2229989	72122794	CT	43	100	0.94[0.65–1.35]			T	43	100	0.94[0.65–1.35]		
			CC	649	1413	ref	–	0.78	C	1341	2926	ref	0.12	0.73
			AA	0	0									
17	rs145813713	72124722	AG	26	45	1.27[0.78–2.08]			A	26	45	1.27[0.78–2.06]		
			GG	666	1468	ref	–	0.36	G	1358	2981	ref	0.92	0.34
			AA	40	81	1.07[0.71–1.60]								
17	rs1042673	72125198	AG	248	536	1.03[0.85–1.24]			A	328	698	1.04[0.89–1.20]		
			GG	404	896	ref	0.24	0.89	G	1056	2328	ref	0.21	0.64
			CC	9	24	0.82[0.38–1.77]								
17	rs74999341	72125313	CT	155	339	1.00[0.80–1.24]			C	173	387	0.97[0.80–1.18]		
			TT	528	1150	ref	0.26	0.88	T	1211	2639	ref	0.07	0.79
			**CC**	**17**	**9**	**1.47[1.18–1.82]**								
**17**	**rs73354570**	**72125316**	**AC**	**155**	**266**	**1.37[1.09–1.71]**			**C**	**189**	**284**	**1.53[1.26–1.86]**		
			**AA**	**528**	**1238**	**ref**	**22.39**	**1.38 × 10**^−**5**^	**A**	**1195**	**2742**	**ref**	**18.09**	**2.11 × 10**^**-5**^

*CHR* chromosome, *POS* position

*Fisher exact tests were performed when sparse cells were presented. Threshold of *P* values was 0.05/7 ≈ 0.007. Significant results were highlighted in bold

**Table 4:** Results of gene by environmental interactions between selected SNPs and smoking and alcoholic drinking

CHR	SNP	POS	Allele	Smoking		Drinking	
				Z.inter	*P*.inter	Z.inter	*P*.inter
17	rs1042673	72125198	A/G	0.49	0.63	− 1.06	0.29
17	rs144221941	72121913	C/T	− 0.34	0.74	− 1.85	0.06
17	rs145813713	72124722	A/G	− 0.51	0.61	0.49	0.62
17	rs2229989	72122794	C/T	1.22	0.22	− 1.42	0.16
17	rs35126550	72122558	A/G	0.80	0.42	1.80	0.07
17	rs73354570	72125316	A/C	− 0.63	0.53	2.34	0.02
17	rs74999341	72125313	C/T	− 0.81	0.42	1.89	0.06

*CHR* chromosome, *POS* position, *Z.inter* Z-statistic of the interaction term, *P.inter P* values of the interaction term

### Bioinformatics analyses

**Fig. 2:**
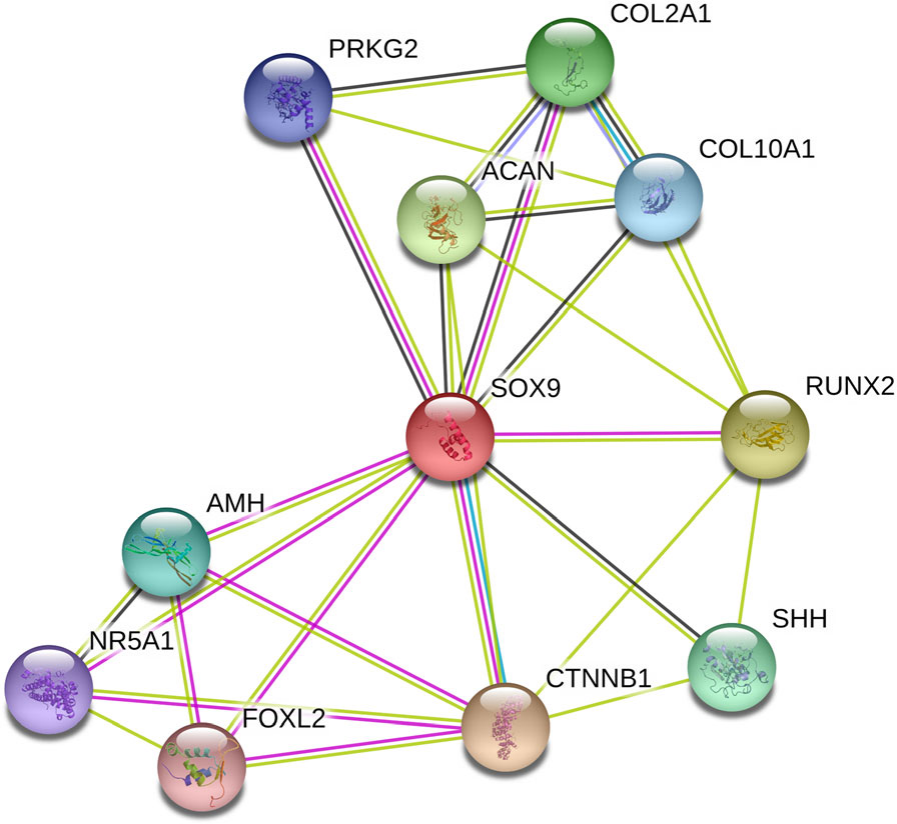
Gene-gene interaction network constructed based on protein-protein interaction (PPI) data. Blue and pink lines mean experimentally determined interactions, and the rest color lines mean the predicted interactions

We explored the potential functional significance of SNP rs73354570 in RegulomeDB and GTEx. The RegulomeDB score for rs73354570 was 2b. The scoring system of RegulomeDB ranges from 1 to 6, and a lower score often indicates that the SNP has a more significant role in biological functions. A further examination of this SNP identified that rs73354570 is located within a region of multiple binding proteins, including CEBPB and POLR2A. This SNP was also part of genetic motifs found within several cell types. We did not identify significant eQTL signals from GTEx for SNP rs73354570 on *SOX9* (Supplemental Table S1). The gene-gene network of *SOX9* is shown in Fig. 2. According to the STRING database, the protein product of *SOX9* interacts experimentally with several proteins encoded by the *PRKG2*, *COL2A1*, *RUNX2*, *AMH*, *NR5A1*, *FOXL2*, and *CTNNB1* genes. In addition, SOX9 was also predicted to be connected with other proteins encoded by genes, including *COL10A1*, *ACAN*, and *SHH*.

## Discussion

In this study, we identified an SNP, i.e., rs73354570, that was significantly associated with the disease status of CTEV in a large Chinese Han population-based sample. To the best of our knowledge, our study is the first to report a significant association between *SOX9* and CTEV. *SOX9* was not reported to be significant in the first and only previous GWAS that focused on clubfoot, which was performed by Zhang et al. [7]. A GWAS with a stringent *P* value threshold (5 × 10^−8^) might miss some true positive hits. Our candidate gene-based study design could avoid this limitation by testing fewer markers of preselected genes. To date, the genes *SOX9* and *SOX9-AS1* (gene encoding anti-sense RNA1 of *SOX9*) have been identified to be associated with several traits and disorders, including angiotensin-converting enzyme inhibitor intolerance [18], height [19], liver enzyme levels (gamma-glutamyl transferase) [20], lung function [21], nose morphology [22], and thyroid hormone levels [23]. However, no GWAS has established the connection between *SOX9* and CTEV-related disorders.

Our bioinformatics analyses have identified that SNP rs73354570 might have significant biological functions in the gene expression of *SOX9*. This SNP would likely affect the protein binding of its surrounding areas and thus might affect the gene expression of *SOX9*. However, this information extracted from RegulomeDB could not be replicated by GTEx eQTL data. No significant differences could be found in the gene expression of *SOX9* in different genotype groups of SNP rs73354570 in multiple human tissues. Thus, the eQTL data extracted from publicly available databases should be interpreted carefully. Gene expression levels could be very different in target tissues of CTEV patients compared to samples collected in GTEx. Therefore, we believe that it is still too early to reach conclusions on the functional significance of the identified SNPs, although there is a good chance that rs73354570 is a surrogate of another underlying marker or a set of markers. No significant signals have been reported in candidate-based association studies. Wang et al. reported that *SOX9* overexpression plays a potential role in idiopathic congenital talipes equinovarus [11]. In the present study, we identified the C allele of rs73354570 to be significantly associated with an increased risk of CTEV. In the future, an eQTL study for rs73354570 on the gene expression of *SOX9* based on the target tissue of CTEV patients should be conducted to evaluate the allelic effects on gene expression.

With the rapid development of sequencing technology, numerous susceptibility loci contributing to complex diseases have been reported, such as schizophrenia [24–26]. Considering that analyses of only some SNPs are not sufficient to draw conclusions [27–31], we conducted gene-by-environment interaction analyses to investigate potential interactions between our selected SNPs and two environmental factors along with genetic association analyses. However, no significant interaction signals were identified. Nevertheless, we believe that it is not necessary to overinterpret this negative result. Since smoking and alcoholic drinking have been proven to be significantly associated with multiple birth defects [32, 33], it is probable that multiple environmental factors could combine with genetic factors to play a role in the process of CTEV onset. Notably, the most significant interaction signal was obtained from the significant hit of association analysis (rs73354570). Although this signal failed to achieve genome-wide significance, it is still worth investigating this interaction signal further in the future because it might partly explain its single marker-based association signal. A potential limitation of the present study is that smoking and drinking status were recorded as habits in general but not status during pregnancy. Additional studies with appropriately measured environmental factors should be conducted to further investigate the combined effects of both environmental factors and genetic factors on CTEV.

Our study has several limitations. The most important factor is the lack of replication. Future studies are needed to replicate our findings regarding *SOX9* in Chinese Han and other populations. Moreover, we did not perform any procedures to adjust for population stratification in the study. However, we tried our best to at least partially control this potential confounding factor because geographic location is a good indicator for genetic matching in the Han Chinese population [34, 35]. Another limitation is that we only included SNPs located within gene regions. However, several important regulatory regions are located at up- or downstream regions that are not within gene regions, and a significant portion of the GWAS panel markers cannot be mapped to any gene regions (but are several kb away from the targeted genes). It is very difficult to claim that seven preselected SNPs could represent most of the genetic information of *SOX9*. In addition, as a candidate gene-based study focusing on the effects of common SNPs, we did not examine any rare or low-frequency variants. However, a recent study showed that low-frequency and rare variants might play an important role in the onset and development of CTEV [36]. Based on Chinese family samples with CTEV, Zhang et al. identified two pathogenic variations from mediator complex subunit 13L (MED13L) and transforming growth factor-β receptor 2 (TGFBR2). Therefore, a sequencing-based study might provide more information about the genetic etiology of CTEV.

In this study, we identified potential links between genetic polymorphisms of *SOX9* and the risk of CTEV. Our study could improve our understanding of the genetic architecture of CTEV and provide a basis for novel intervention plans. Replication studies involving Chinese Han and other populations are still needed in the future.

## Supplementary information

**Additional file 1: Table S1.** eQTL signals for SNP rs73354570 on gene SOX9.

## Data Availability

Please contact the authors for reasonable requests.

## Abbreviations

CTEV: Congenital talipes equinovarus
GWAS: Genome-wide association study
MAF: Minor allele frequency
HWE: Hardy-Weinberg equilibrium
LD: Linkage disequilibrium
eQTL: Expression quantitative trait loci
ORs: Odds ratios
SNP: Single-nucleotide polymorphism
