# Supplementary material for: Evaluation of genetic susceptibility of common variants in SOX9 in patients with congenital talipes equinovarus in the Han Chinese population
Source: J Orthop Surg Res. 2020 Jul 23;15:276. doi: 10.1186/s13018-020-01802-7 (PMC7376870; doi:10.1186/s13018-020-01802-7)
Supplement: Supplementary file 1 — Additional file 1: Table S1. eQTL signals for SNP rs73354570 on gene SOX9. [file 13018_2020_1802_MOESM1_ESM.doc]

Supplemental Table S1. eQTL signals for SNP rs73354570 on gene *SOX9*.

| Gene | SNP | *P*-Value | NES | *T*-statistic | Tissue |
| --- | --- | --- | --- | --- | --- |
| *SOX9* | rs73354570 | 0.002 | -0.620 | -3.10 | Adrenal Gland |
| *SOX9* | rs73354570 | 0.029 | -0.250 | -2.20 | Heart - Left Ventricle |
| *SOX9* | rs73354570 | 0.040 | 0.220 | 2.10 | Brain - Nucleus accumbens (basal ganglia) |
| *SOX9* | rs73354570 | 0.041 | 0.230 | 2.10 | Adipose - Visceral (Omentum) |
| *SOX9* | rs73354570 | 0.065 | 0.190 | 1.90 | Brain - Cerebellum |
| *SOX9* | rs73354570 | 0.078 | -0.180 | -1.80 | Artery - Tibial |
| *SOX9* | rs73354570 | 0.080 | -0.170 | -1.80 | Brain - Caudate (basal ganglia) |
| *SOX9* | rs73354570 | 0.160 | -0.200 | -1.40 | Artery - Aorta |
| *SOX9* | rs73354570 | 0.190 | -0.120 | -1.30 | Brain - Cortex |
| *SOX9* | rs73354570 | 0.190 | -0.180 | -1.30 | Colon - Sigmoid |
| *SOX9* | rs73354570 | 0.200 | 0.180 | 1.30 | Brain - Substantia nigra |
| *SOX9* | rs73354570 | 0.210 | 0.160 | 1.30 | Brain - Amygdala |
| *SOX9* | rs73354570 | 0.240 | -0.140 | -1.20 | Heart - Atrial Appendage |
| *SOX9* | rs73354570 | 0.240 | 0.320 | 1.20 | Kidney - Cortex |
| *SOX9* | rs73354570 | 0.260 | -0.190 | -1.10 | Artery - Coronary |
| *SOX9* | rs73354570 | 0.280 | -0.130 | -1.10 | Esophagus - Muscularis |
| *SOX9* | rs73354570 | 0.330 | 0.130 | 0.98 | Cells - Cultured fibroblasts |
| *SOX9* | rs73354570 | 0.360 | -0.094 | -0.92 | Lung |
| *SOX9* | rs73354570 | 0.410 | 0.066 | 0.82 | Breast - Mammary Tissue |
| *SOX9* | rs73354570 | 0.540 | -0.190 | -0.62 | Uterus |
| *SOX9* | rs73354570 | 0.560 | 0.065 | 0.59 | Brain - Anterior cingulate cortex (BA24) |
| *SOX9* | rs73354570 | 0.560 | 0.032 | 0.59 | Skin - Not Sun Exposed (Suprapubic) |
| *SOX9* | rs73354570 | 0.580 | 0.068 | 0.55 | Prostate |
| *SOX9* | rs73354570 | 0.600 | -0.067 | -0.52 | Adipose - Subcutaneous |
| *SOX9* | rs73354570 | 0.650 | 0.037 | 0.46 | Colon - Transverse |
| *SOX9* | rs73354570 | 0.670 | 0.073 | 0.43 | Pancreas |
| *SOX9* | rs73354570 | 0.680 | 0.071 | 0.41 | Cells - EBV-transformed lymphocytes |
| *SOX9* | rs73354570 | 0.700 | -0.051 | -0.39 | Brain - Hypothalamus |
| *SOX9* | rs73354570 | 0.700 | 0.023 | 0.39 | Testis |
| *SOX9* | rs73354570 | 0.710 | 0.037 | 0.38 | Stomach |
| *SOX9* | rs73354570 | 0.730 | -0.033 | -0.34 | Thyroid |
| *SOX9* | rs73354570 | 0.740 | 0.100 | 0.33 | Liver |
| *SOX9* | rs73354570 | 0.750 | 0.035 | 0.32 | Brain - Putamen (basal ganglia) |
| *SOX9* | rs73354570 | 0.780 | 0.071 | 0.28 | Brain - Spinal cord (cervical c-1) |
| *SOX9* | rs73354570 | 0.780 | 0.018 | 0.28 | Skin - Sun Exposed (Lower leg) |
| *SOX9* | rs73354570 | 0.820 | 0.025 | 0.23 | Brain - Hippocampus |
| *SOX9* | rs73354570 | 0.830 | 0.035 | 0.21 | Minor Salivary Gland |
| *SOX9* | rs73354570 | 0.830 | -0.052 | -0.22 | Ovary |
| *SOX9* | rs73354570 | 0.860 | -0.017 | -0.18 | Brain - Cerebellar Hemisphere |
| *SOX9* | rs73354570 | 0.860 | 0.018 | 0.18 | Esophagus - Mucosa |
| *SOX9* | rs73354570 | 0.870 | -0.014 | -0.16 | Brain - Frontal Cortex (BA9) |
| *SOX9* | rs73354570 | 0.880 | 0.024 | 0.16 | Pituitary |
| *SOX9* | rs73354570 | 0.930 | 0.008 | 0.09 | Small Intestine - Terminal Ileum |
| *SOX9* | rs73354570 | 0.940 | 0.018 | 0.08 | Spleen |
| *SOX9* | rs73354570 | 0.960 | 0.014 | 0.06 | Vagina |
| *SOX9* | rs73354570 | 0.970 | -0.003 | -0.04 | Nerve - Tibial |
| *SOX9* | rs73354570 | 0.980 | 0.002 | 0.02 | Muscle - Skeletal |

NES: normalized effect size.
